# Hormone Replacement Therapy in a Patient with Hypogonadism and Coexisting Medical Conditions

**DOI:** 10.4274/jcrpe.galenos.2020.2019.S0021

**Published:** 2020-02-06

**Authors:** Özlem Dural, Şükran Poyrazoğlu

**Affiliations:** 1 İstanbul University, İstanbul Faculty of Medicine, Department of Obstetrics and Gynecology, İstanbul, Turkey; 2 İstanbul University, İstanbul Faculty of Medicine, Unit of Pediatric Endocrinology, İstanbul, Turkey

**Keywords:** Adolescent, hypogonadism, hormone replacement therapy

## Abstract

In adolescents and young women, there is limited data on the type of replacement, route of administration, and ideal doses to be used in systemic hormone therapy administered for the treatment of hypogonadism. In particular, management of patients with complicated systemic diseases or at risk of thrombophilia may present significant challenges. We present a case of a 15-year-old adolescent girl with hypogonadism and coexisting medical conditions, who was evaluated for systemic hormone therapy.

## Introduction

Systemic hormone therapy in adolescents and young women with hypogonadism is an effective treatment for the symptoms of hypoestrogenism, thus reducing long-term health risks. Oral or transdermal hormone replacement therapy (HRT) that provides the physiological hormone levels required for this age group is considered as a first-line approach, although combined oral contraceptives (COCs) can be also used for estrogen replacement (1,2). We report a 15 year-old girl diagnosed with hypogonadotropic hypogonadism, who also had obesity, dyslipidemia, factor V Leiden mutation and a history of renal transplantation. We present this case with the aim of discussing the approach and appropriate treatment in terms of systemic hormone therapy.

## Case Report

A 15.3 year-old girl presented with short stature and primary amenorrhea. She had normal birth weight at 40 weeks of gestation. She had chronic renal failure due to nephrotic syndrome since the age of two years and had renal transplantation two years prior to this presentation. She was on glucocorticoid and immunosuppressive drugs (micofenolat, mofetil and tacrolimus) and was on enalapril for hypertension. Her parents were unrelated and family history was unremarkable.

On physical examination, her weight was 46.6 kg [1.6 standard deviation score (SDS)] and height 129.4 cm (-5.5 SDS) and body mass index was 27.7 kg/m^2^ (2.1 SDS). She had central obesity, a dorsocervical fat pad, hirsutism and striae. Her blood pressure was 110/80 mmHg. The rest of the physical examination was normal. She had undergone normal puberty without menarche and breast and pubic hair were at Tanner stage 5.

Laboratory tests including complete blood count, glucose, electrolytes, calcium profile, prothrombin time, activated partial thromboplastin time, renal and liver function tests were within normal range. She had dyslipidemia (total cholesterol=248 mg/dL, low-density lipoprotein cholesterol=168 mg/dL). Other laboratory results were normal, including thyroid function tests, prolactin concentration, parathyroid hormone and 25-hydroxy vitamin D (Table 1). She had osteoporosis on dual energy X-ray absorbtiometry (DXA; L1-L4 Z score-2).

She had low luteinizing hormone, follicle-stimulating hormone and estradiol concentrations (Table 1). Pelvic ultrasound was normal (right ovary volume 7 mL, left ovary volume 7.2 mL with normal echogenicity and uterus volume 49.2 mL). Thrombophilia investigation of the patient showed a heterozygous factor V Leiden mutation. Other investigations for thrombophilia were within normal ranges. Final diagnoses of this patient with kidney transplant were pubertal arrest, hypogonadotrophic hypogonadism, obesity, dyslipidemia, osteoporosis and factor V Leiden heterozygous mutation.

There are some clinical risks evident for the management of this patient. The patient would need HRT for hypogonadism and osteoporosis but had an increased risk of thromboembolism due to the co-existence of factor V Leiden heterozygous mutation and obesity. Thus tailoring the HRT therapy, including the product, dose and route of administration in this patient to avoid some side effects of treatment, was needed. Taking into consideration the existing medical conditions and the increased risk of thromboembolism, she was started on transdermal estrogen treatment (100 micrograms 17 beta estradiol daily) with cyclic oral progesterone replacement (10 mg dydrogesterone for the first 12 days of each month). No side effects or complications were encountered during the first year of treatment.

## Discussion

The purpose of estrogen replacement in adolescent and young women with hypogonadism is both to treat the symptoms of hypoestrogenism and to mitigate long term health risks, such as osteoporosis and cardiovascular disease. Although the data on optimal HRT for these patients are limited, hormone replacement (either orally or transdermally) that provides the physiological hormone levels required for this age group should be the first choice treatment (1,2). Estrogen replacement can be also achieved with COCs. However, COCs contain ethinyl estradiol, a synthetic estrogen that is more potent than 17 beta estradiol included in HRT preparations. In addition, they contain higher dose progestins, which maintain primary contraceptive activity. Therefore, they provide more steroid hormone than is needed for physiologic replacement, with unfavourable adverse effects on lipid profile, blood pressure and on haemostatic factors (3) and, at the same time, the effect on bone density is less favorable compared with HRT (4,5,6). For these reasons, HRT is preferrable to COCs for the treatment of hypogonadism compare; HRT is considered the first choice.

The most commonly used estrogen preparations for HRT and recommended dosages are 2 mg oral or 100 micrograms transdermal 17 beta estradiol daily (1,2). To prevent the development of endometrial pathologies in patients with intact uterus, estrogen replacement should be combined with the appropriate dose of progestins. There is also a lack of evidence on the effect of various progestogen preparations in HRT for reproductive age women and adolescents with hypogonadism. However, evidence from postmenopausal women appears to favor micronized natural progesterone due to a better cardiovascular profile and possible risk reduction in breast cancer (7,8). For effective endometrial protection, 10 mg medroxyprogesterone acetate or 200 mg micronized oral progesterone for a minimum of 10 to 12 days per month is required in sequential treatments (9).

Primary or secondary amenorrhea associated with hypoestrogenism affects the acquisition and maintenance of peak bone mass in young women and it is associated with both the degree and duration of estrogen-deficiency (10,11,12). In contrast to postmenopausal women, low bone mass in these young patients is managed most appropriately with HRT instead of antiresorptive drugs such as bisphosphonates (1). Although COCs are sometimes used for estrogen replacement in women with hypogonadism, it has been shown that HRT is superior to COC in increasing bone density at the lumbar spine in women with premature ovarian insufficiency or functional hypothalamic amenorrhea (4,5,6). Compared to the oral route, the transdermal administration of estrogen provides higher and more consistent plasma levels of estradiol, and also does not have a negative effect on serum insulin-like growth factor-1 level, due to avoidance of the ‘first past’ effects on the liver (13,14). Therefore, transdermal administration may be preferable for HRT in women with severe osteoporosis. In addition, transdermal HRT also appears to have a beneficial effect on serum lipid profiles, inflammatory markers, and blood pressure (15).

Oral estrogen therapy is associated with an increased risk of venous thromboembolism (VTE) because of its effect on the balance between procoagulant factors and antithrombotic mechanisms (16). Since estrogen replacement doses provided in HRT are less potent than estrogen in COC, HRT is expected to carry a lower risk of VTE. Current evidence of VTE risk in HRT users with menopause at a regular age has shown an increased risk (16). Although there is no data on HRT use and VTE risk in adolescents and young adults with hypogonadism, given that increased age is an important risk factor for VTE, there is an assumption that increase in VTE risk associated with HRT use will be lower than in the postmenopausal population. To further reduce the risk of VTE with HRT, the transdermal route should be recommended for elimination of the ‘first past’ effects on the liver. Even in studies on HRT users with menopause at the expected age, increased risk of thrombosis has not been shown with transdermal estrogen replacement. In addition, transdermal estrogen does not confer an additional risk in women who carry a prothrombotic mutation (17,18,19). Therefore, transdermal estrogen replacement appears to be a reasonable approach in young women, who have hypogonadism but are at risk of VTE, including patients carrying a prothrombotic mutation. In terms of progestogens used in hormone replacement and VTE risk, no risk increase was shown with the use of micronized progesterone and pregnane derivatives, such as dydrogesterone, medroxyprogesterone acetate and cyproterone acetate, while studies showing an increased in risk with the use of norpregnane derivatives, such as nomegestrol acetate or promegestone, are available (17,19).

In our case, a 15 year-old girl with renal transplant, obesity, dyslipidemia, factor V Leiden mutation, osteoporosis and hypogonadotropic hypogonadism was evaluated for HRT. Factor V Leiden mutation is one of the two most common genetic defects associated with an increased risk of VTE and leads to a 4-5-fold increase in VTE risk (20,21). Taking into consideration the existing medical conditions and the increased risk of VTE in our patient, it was decided to initiate transdermal estrogen treatment with cyclic oral dydrogesterone. Again, there is little evidence concerning endometrial protection in adolescents and young adults, but dydrogesterone appears to be a safe progestogen with an acceptable metabolic profile, similar to micronized progesterone (22).

In contrast to the mild increase in VTE risk present in our patient, patients with hypogonadism but at high-risk of VTE, including patients with high-risk prothrombotic mutations such as homozygous factor V Leiden mutation or antithrombin-3 deficiency, may still benefit from HRT. In the management of such patients, it seems prudent to consult with a hematologist before starting HRT, since prophylactic anticoagulation therapy may be required.

## Figures and Tables

**Table 1:**
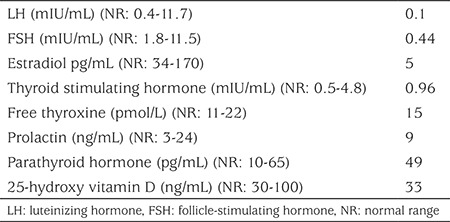
Hormone analysis of the patient
